# A prediction model to differentiate transient ischemia from irreversible transmural necrosis in closed-loop small bowel obstruction

**DOI:** 10.3389/fmed.2024.1466754

**Published:** 2024-09-11

**Authors:** Shaorong Pan, Jiejin Yang, Zining Liu, Rile Nai, Zeyang Chen

**Affiliations:** ^1^Department of Gastrointestinal Surgery, Peking University First Hospital, Peking University, Beijing, China; ^2^Department of Radiology, Peking University First Hospital, Peking University, Beijing, China; ^3^Department of Gynecologic Oncology, National Cancer Center/National Clinical Research Center for Cancer/Cancer Hospital, Chinese Academy of Medical Sciences & Peking Union Medical College, Beijing, China

**Keywords:** closed-loop small bowel obstruction, bowel ischemia, bowel necrosis, computed tomography, diagnostic model

## Abstract

**Introduction:**

The study aims to develop a prediction model to differentiate transient ischemia from irreversible transmural necrosis in closed-loop small bowel obstruction (CL-SBO).

**Methods:**

A total of 180 participants with CL-SBO between January 2010 and December 2019, of which 122 had complete radiologic data, were included to investigate the significant clinical and imaging characteristics for differentiating patients with necrosis from transient ischemia. A logistic regression model was developed and validated.

**Results:**

In a multivariate analysis, the American Society of Anesthesiologists physical status classification system >2 is the independent predictor for transmural necrosis. Another multivariate analysis, including clinical and imaging factors, revealed that the imaging sign of mesenteric vessel interruption was an independent risk factor for necrosis. The diagnostic model developed using these two factors has excellent performance among the validation sets, with an area under the curve of 0.89.

**Conclusion:**

The diagnostic model and innovative imaging signs have substantial potential in solving this complex clinical problem.

## 1 Introduction

Small bowel obstruction (SBO) is one of the most common acute abdomen diseases, with approximately 96,307 and 45,178 cases reported in China and the USA, respectively, according to the Global Burden of Disease Study 2019 (1). About 10% of SBO patients with intestinal ischemia are classified as strangulated small bowel obstruction (SSBO). SSBO is primarily caused by closed-loop small bowel obstruction (CL-SBO), which involves the mechanical obstruction of a bowel loop at two adjacent points and compression of the vascular pedicle (2–4). Internal hernia and adhesive bands are common causes of CL-SBO. CL-SBO typically leads to two distinct outcomes: transient ischemia and transmural necrosis (5). Most patients diagnosed with CL-SBO should undergo emergent surgical exploration to avoid life-threatening complications (2, 6), although some highly selected patients may benefit from non-surgical management (7, 8). The blood supply for bowel loops can be restored for transient intestinal ischemia after releasing the obstruction during the operation. Conversely, necrotic bowel segments must be resected for transmural necrosis (8, 9). Transmural necrosis is one of the most severe consequences of SBO and needs emergency treatment as soon as possible to reduce lethal complications (3, 10). Unlike laparoscopic adhesiolysis, which is suitable for some patients with transient intestinal ischemia, transmural necrosis carries a high risk of diffuse peritonitis, septic shock, and bowel perforation, making it a relative contraindication for laparoscopic surgery (11). The severity and process flow differences make preoperative differentiation of transient ischemia from transmural necrosis essential among CL-SBO patients.

Some researchers focus on clinical characteristics, including medical history, clinical symptoms, physical examination results, and laboratory indicators, which unfortunately lack sufficient sensitivity and specificity to accurately distinguish between transient ischemia and necrosis in SSBO patients (12). In terms of imaging examinations, there is a consensus on the use of computed tomography (CT), which can comprehensively provide information on the cause, location and extent of obstruction when evaluating patients with SBO, especially with unenhanced CT, known for its convenience and efficiency (2, 13). However, when diagnosing CL-SBO, CT demonstrates suboptimal diagnostic performance, with a sensitivity of 53% and specificity of 83% (14). The unsatisfactory performance of clinical or CT features in diagnosing SBO subtypes prompts reconsideration of preoperative differentiation among the various subtypes of CL-SBO (15–18). This study explores significant clinical and CT features that can accurately identify transient ischemia and transmural necrosis of CL-SBO prior to surgery, potentially aiding surgeons in developing more timely and comprehensive operative plans.

## 2 Materials and methods

### 2.1 Patient selection and data collection

The Institutional Ethics Committee of Peking University First Hospital approved this retrospective study, and informed consent was obtained from all participants. Eight hundred thirty-three patients diagnosed with intestinal obstruction who underwent surgery between January 2010 and August 2022 were identified from the institution’s database. CL-SBO was defined as mechanical obstruction of a bowel loop at two adjacent points, accompanied by compression of the vascular pedicle. The final diagnosis of CL-SBO was made intraoperatively, with terms such as “closed loop obstruction” or “multiple zones of transition” documented in the surgical report. The following exclusion criteria were applied: (a) Patients with colorectal obstruction; (b) Patients with an abdominal external hernia; (c) Patients with small bowel obstruction without a closed loop, including vascular occlusion-associated SBO, simple adhesive SBO, paralytic ileus, or obstruction caused by space-occupying lesions (e.g., tumor, bezoar, polyp) or intussusception. Ultimately, 242 patients with colorectal obstruction, 47 with an abdominal external hernia, 26 with space-occupying lesions in the small intestine, 257 with simple adhesive SBO, and 31 with vascular intestinal obstruction were excluded, leaving 230 participants with CL-SBO in the study. The classification of CL-SBO was confirmed during the operation. Transmural necrosis was confirmed when the dark purple-colored intestinal segment did not return to its normal color within 20 min after surgical release of the obstruction, as previously described (2), and was further confirmed by histologic analysis. The study included patients with CL-SBO between January 2010 and August 2022, including 118 patients with transmural necrosis who underwent partial enterectomy, and 112 with transient ischemia relieved after the obstruction was cleared. The screening process for this study is shown in Supplementary Figure S1.

Clinical and demographic parameters, including gender, age, body mass index (BMI), American Society of Anesthesiologists (ASA) physical status classification system, admission symptoms (abdominal pain, vomiting, exhaust or defecation), vital signs, history of abdominal surgery or infection, preoperative stomach tube placement, preoperative anti-inflammatory treatment, time from onset to operation (hours), percentage of neutrophils, percentage of lymphocytes, and the neutrophil-to-lymphocyte ratio (NLR) were considered. These data were collected by the surgeon S.P.

### 2.2 CT acquisition

Between January 2010 and December 2019, one hundred and twenty -two (122/180) patients have complete radiographic data from our institution, with the interval between CT and surgery being within 24 h. For patients between January 2020 and August 2022, 50 patients included in this study also had complete radiographic data from our institution, with the CT – surgery interval within 24 h. All of these patients underwent unenhanced CT. The CT examinations were performed using multislice spiral CT machines, including the Philips Brilliance 64 or Philips Brilliance iCT (Philips Medical Systems, Cleveland, OH, USA); GE Discovery CT750 m or GE LightSpeed VCT (GE Healthcare, Princeton, NJ, USA); Siemens Somatom Sensation 64 (Siemens, Forchheim, Germany). The spiral CT parameters were: 120 kV tube voltage, 150–250 mA tube current, 0.5–0.8 s tube rotation time, 64 × 0.625 mm detector collimation, 350 × 350 mm field of view, 512 × 512 matrices, 1.25 mm section thickness and 1 mm reconstruction interval.

In daily clinical practice, our group observed that discontinuous mesenteric vessels (arterial and venous vessels irrespective), where mesenteric vessels abruptly truncate at certain points, and the proximal portion of the mesenteric vessels distends from retrograde or collateral inflows in the strangulated intestine on CT images, might be significant in differentiating CL-SBO. This imaging sign was defined as the “mesenteric vessel interruption,” which has previously only been reported in the superior mesenteric vein (19). Based on the previous literatures (3, 20–24), other CT findings were also selected for analysis in our study: (a) Increased unenhanced bowel-wall attenuation, defined as an increase in attenuation of the wall of the dilated loop compared to adjacent typical bowel loops on unenhanced images (25); (b) Bowel wall thickening, defined as a thickness greater than 3 mm; (c) Bowel loop dilatation, defined as a diameter greater than 25 mm; (d) Mesenteric fluid, defined as fluid in the mesentery of the affected intestine; (e) Mesenteric haziness, defined as increased attenuation of the mesenteric fat, which can be graded as absent, focal (only in the involved loop) or diffuse (the mesentery beyond the obstructed bowel segment is involved); (f) Mesenteric venous engorgement, defined as dilation of the mesenteric veins of the obstructed bowel compared to neighboring loops; (g) Radial distribution, defined as mesenteric vessels distributed radially; (h) Pneumatosis intestinalis, defined as intramural gas; (i) Pneumoperitoneum, defined as free peritoneal gas; (j) Peritoneal fluid, graded as absent, small (confined to the pelvic cavity or peritoneal cavity) or large (overflowed into the perihepatic and perisplenic spaces); (k) Whirl sign, defined as a swirled appearance of the mesenteric vessels and fat, reflecting the rotation of the mesentery; (l) Feces sign, defined as a mix of particulate matter with gas bubbles within the dilated bowel, classified as proximal to the obstruction site (FS1), within the closed loop (FS2) or at both sites (FS3); (m) Degree of obstruction, graded as high (completely occlusive loop) or low (incompletely occlusive loop). CT images were reviewed indenpendently by two experienced radiologists (each with over 5 years of experience in abdominal CT) who were blind to the patients’ surgically confirmed diagnosis and clinical parameters. For cases where there was initial disagreement, the two radiologists reached a consensus through discussion.

### 2.3 Statistical analysis

Firstly, univariate analysis using *t*-test, chi-square test, Wilcoxon rank sum test and Fisher’s exact test was performed to compare clinical characteristics between the transmural necrosis and transient ischemia group for patients between January 2010 and December 2019. Variables with *p* < 0.05 were included in the multivariate analysis using logistic regression to select an independent predictor for transmural necrosis with a two-sided *p* value <0.05. The odds ratios (OR) and 95% confidence intervals (CIs) were calculated. As there may not be many independent predictors, variables with *p* < 0.10 were used to develop a multivariate logistic regression model for outcome prediction. The predicted ability of the model was measured using Harrell’s concordance index (C-index), in which the predicted values were deduced using the multivariate logistic regression formula: predictive value = $\overset{n}{\underset{i=1}{\sum }}\beta i\times \mathrm{Xi}$. Internal validation of the prediction model was assessed using the bootstrapping method. The validation cohort used patients’ data from January 2020 to August 2022. An acceptable C-index was equal to or higher than 0.7. The receiver operation characteristic (ROC) curve was used to visualize the discriminatory power of the prediction model.

## 3 Results

### 3.1 Association of clinical and CT features with transmural necrosis

During the univariate analysis of clinical characteristics in all patients between January 2010 and December 2019 (Supplementary Table A), patients with transmural necrosis (56/96, 58.3%) have a significantly higher proportion of advanced age, exceeding 65 years, than the transient ischemia group (36/84, 42.9%, *p* = 0.038). Patients with an ASA score greater than or equal to 3 are remarkably more common in the transmural necrosis group (44/96, 45.8% versus 21/84, 25%, *p* = 0.004). Regarding routine blood examination indices, the transmural necrosis group has significantly higher NLR (9.7 versus 7.8, *p* = 0.044) and lower lymphocyte percentage (8.9% versus 10.5%, *p* = 0.045) than participants in the transient ischemia group. The multivariate analysis data (Supplementary Table B) indicate that only the ASA score was an independent risk factor for transmural necrosis in patients with CL-SBO (*p* = 0.049, OR = 2.103).

**Figure 1:**
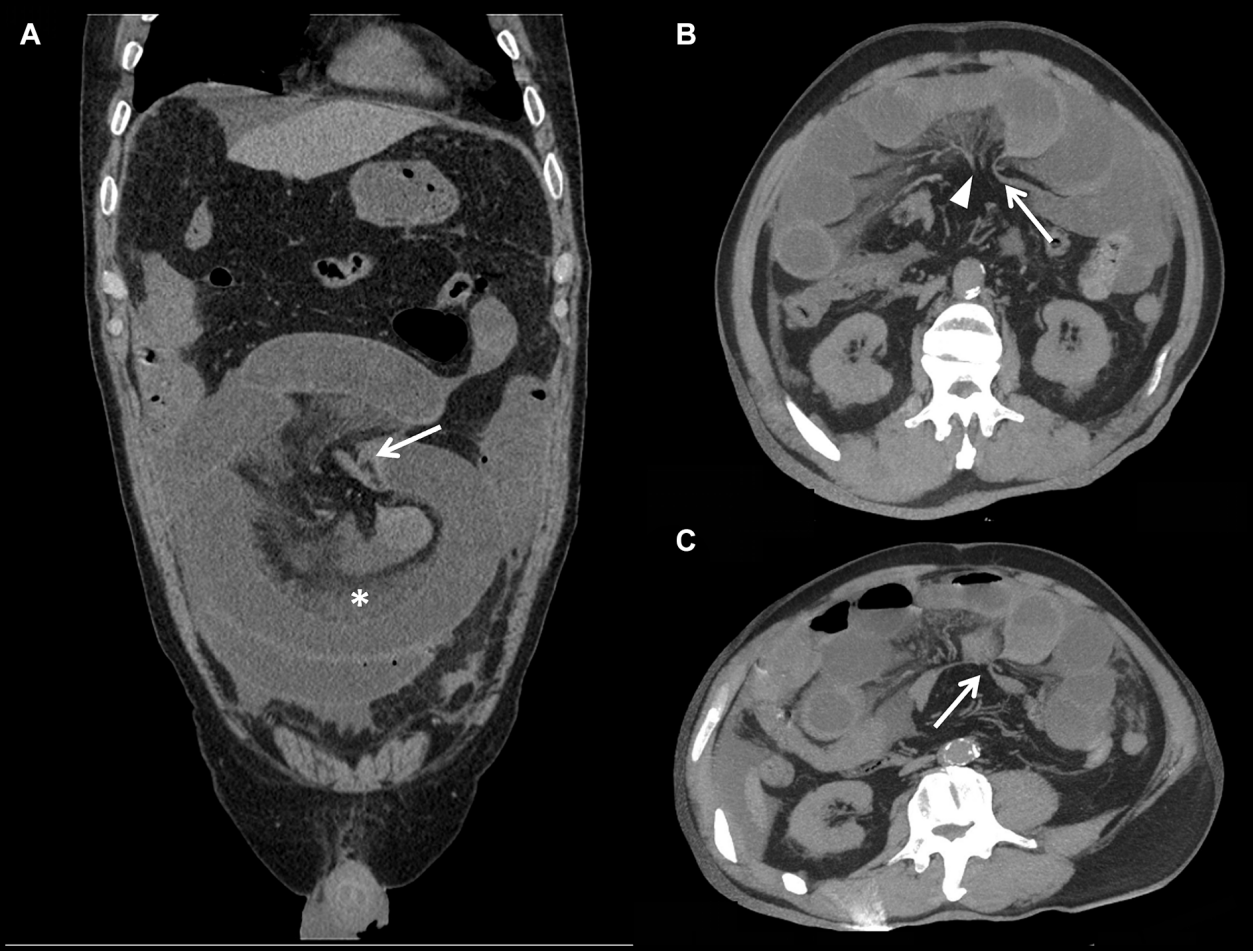
Non-enhanced CT images in 59-year-old man show strangulated ileus, transmural necrosis of the small bowl wall due to internal hernia. Panel **(A)** is MPR (multi-planar reconstruction) image. Panels **(B,C)** are MIP (Maximum intensity projection) images reconstructed from different angles. The images show that the small intestine herniated into the greater omentum is dilated with fluid accumulation, and the attenuation of the intestinal wall is increased. The white arrow points to the site of intestinal strangulation. Mesenteric vessels are interrupted at the site of strangulation (arrowhead), and proximal mesenteric vessels are tortuous and dilated. Simultaneously, the mesenteric haziness (*) was shown.

**Figure 2:**
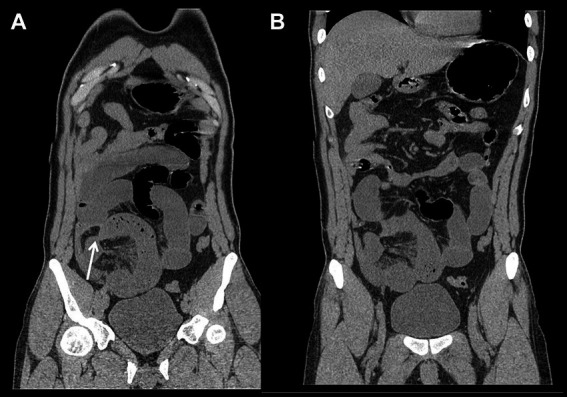
A 36-year-old female with surgically confirmed transient intestinal ischemic obstruction caused by small bowel volvulus in the right lower quadrant. The MPR images **(A,B)** show a dilated small bowl loop in the right lower quadrant with a vortex-like distribution. The position indicated by the arrow shows sharp narrowing of the small intestine. **(B)** Shows the widen and uninterrupted of vessels in the mesenteric combining with misty mesentery sign.

In the subgroup analysis, the univariate analysis (Supplementary Table C) also suggests that a higher ASA score was more commonly seen in patients with transmural necrosis (26/57, 45.6% versus 18/65, 27.7%, *p* = 0.041). For the CT features, univariate analysis (Supplementary Table D) reveals that positive increased unenhanced bowel-wall attenuation (15/57, 26.3% versus 4/65, 6.2%, *p* = 0.003), bowel wall thickening (37/57, 64.9% versus 21/65, 32.3, *P* < 0.001), mesenteric fluid (47/57, 82.5% versus 39/65, 60%, *p* = 0.007), diffuse mesenteric haziness (38/57, 66.7% versus 30/65, 46.2%, *p* = 0.017) and mesenteric vessel interruption (41/57, 71.9% versus 20/65, 30.8%, *P* < 0.001) are significantly more common in the transmural necrosis than transient ischemia group. The logistic regression outcomes (Table 1) indicate that mesenteric vessel interruption (OR = 4.015, *p* = 0.002) is an independent risk factor for transmural necrosis. The CT images of typical cases are shown in Figures 1, 2.

**Table 1:** Independent risk factor analysis of intestinal resection in subgroup.

Variables	Multivariate analysis	
	Odds Ratio (95%CI)	*p*-value
ASA score	2.293 (0.923–5.698)	0.074
Increased unenhanced bowel-wall attenuation	1.958 (0.477–8.031)	0.351
Bowel wall thickening	2.197 (0.840–5.750)	0.109
Mesenteric fluid	1.906 (0.644–5.642)	0.244
Mesenteric haziness	0.807 (0.348–1.872)	0.617
Peritoneal fluid	1.348 (0.569–3.197)	0.498
Feces sign	0.862 (0.518–1.436)	0.569
Interruption of the mesenteric vessel	4.015 (1.669–9.655)	**0.002**

ASA, American Society of Anesthesiologists. The bold values indicate *p* value < 0.05.

### 3.2 Model development

**Figure 3:**
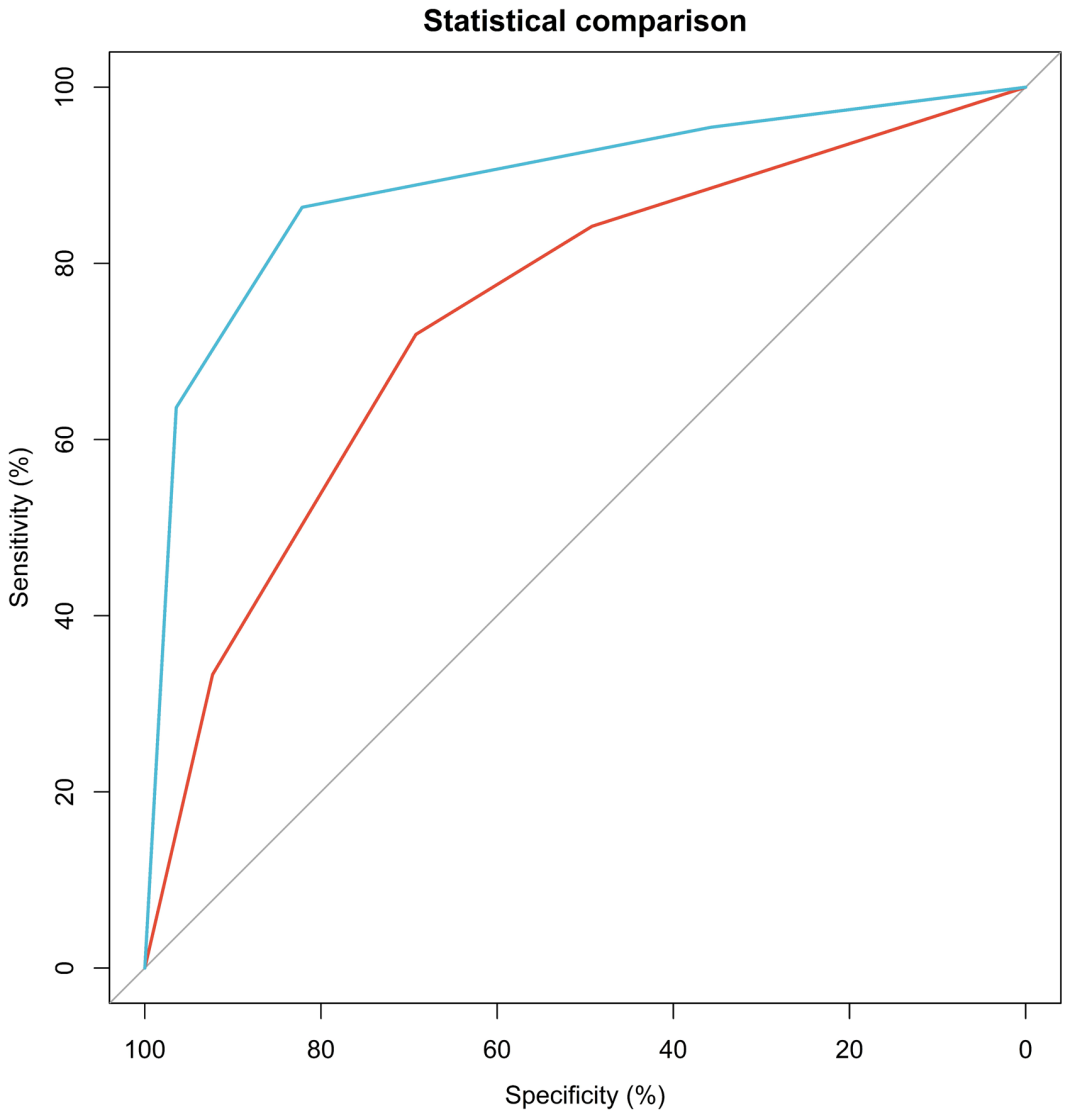
ROC curve for four tiers performances in training and validation groups.

According to the multivariate analysis, only ASA > 2 and mesenteric vessel interruption achieved *p*-values under 0.10 and so were used for model construction. The predictive value was “0.802 × ‘ASA status’ + 1.76 × ‘mesenteric vessel interruption status’.” Since both ASA and mesenteric vessel interruption are dichotomous variables, the multivariate logistic model can be regarded as a four-tier classifier ranking from “ASA ≤ 2 and interruption (−)” (lowest risk group) to “ASA > 2 and interruption (+)” (highest risk group). The C-index of this four-tier classification is 0.744 (0.659–0.829) in the training cohort (*N* = 122). The calibration curve shown in Supplementary Figure S2 is close to the diagonal line within 45 degrees, indicating a well-calibrated predictive model. The C-index is 0.890 (0.794–0.985) in the external validation group, even higher than in the training group (Figure 3). The sensitivity, specificity, PPV, NPV and accuracy under each cutoff point in the training and validation cohorts are shown in Tables 2, 3. For patients with ASA > 2 and interruption positivity, the specificity was 92.31 and 96.43% in training and validation cohorts, respectively, which indicating a low misdiagnosis rate. Meanwhile, the sensitivity increased to 84.21% (78.05% NPV) in the training group and to 95.45% (90.91% NPV) in the validation group when we “used interruption (−) and ASA ≤ 2″. The maximum accuracy was achieved when interruption status was used as the cutoff in both the training and validation cohorts. Different cutoff points provide different strategies under different clinical backgrounds. The establishment of this model predicts the surgical risk and greatly influences the choice of treatment strategies. Patients in the highest risk group are more likely to develop bowel necrosis. Therefore, more attention should be paid to the changes of these patients’ conditions in emergency diagnosis and treatment. Anti-infection and shock prevention therapy were actively administered, and the preferred surgical approach was open surgery rather than laparoscope. For patients in the lowest risk group, conservative treatment can be tried.

**Table 2:** Sensitivity, specificity, PPV and NPV at given cutoff point in training set.

	Sensitivity	Specificity	Positive predictive value (PPV)	Negative predictive value (NPV)	Accuracy
Interruption (+) and ASA > 2	33.33%	92.31%	79.17%	61.22%	64.75%
Interruption (+)	71.93%	69.23%	67.21%	73.77	70.49%
Interruption (−) and ASA ≤ 2	84.21%	49.23%	59.26%	78.05%	65.57%

**Table 3:** Sensitivity, specificity, PPV and NPV at given cutoff point in validation set.

	Sensitivity	Specificity	Positive predictive value (PPV)	Negative predictive value (NPV)	Accuracy
Interruption (+) and ASA > 2	63.64%	96.43%	93.33%	77.14%	82.00%
Interruption (+)	86.36%	82.14%	79.17%	88.46%	84.00%
Interruption (−) and ASA ≤ 2	95.45%	35.71%	53.85%	90.91%	62.00%

## 4 Discussion

CL-SBO is a common acute abdomen disease that usually requires surgical intervention. Considering this fact, an accurate operational strategy is essential in treating patients with CL-SBO, such as using laparoscopy and evaluating patients’ critical degree (16, 26). The abdominal CT is a convenient and fast preoperative examination widely applied in diagnosing variable acute abdomens, including SBO (2, 3). Differentiation SSBO from simple intestinal obstruction using clinical and imaging signs is necessary for surgeons and radiologists, which may become an indication for supporting conservative treatment application (13). Many studies also explore significant clinical and radiological signs for differentiating patients with SSBO, such as reduced bowel wall enhancement, a closed-loop mechanism, diffuse mesenteric haziness and so on (3, 14, 21, 22, 24, 27). In contrast, as the most common type of SSBO, CL-SBO has few physicians and studies focusing on its degree of ischemia, transient ischemia and transmural necrosis, which may also play a decisive role in establishing the surgical strategy (2, 28).

The current study screened some significant clinical characteristics from CL-SBO patients with transient ischemia or transmural necrosis, such as advanced age, ASA score and NLR. Some clinical symptoms and signs, such as peritoneal irritation, did not reveal a significant difference between the ischemic and necrotic groups. Patients with transmural necrosis have higher NLR, which can reflect the severity of systemic inflammation (29). Like this study, Yoon et al. also indicated that NLR increased significantly in SBO patients with poor prognoses (30). Tim et al. suggested that some common symptoms or signs in the acute abdomen had limited diagnostic value in SBO patients with different intestinal blood supply statuses (31). Whether in total patients or subgroup analysis, the higher ASA classification was the risk factor for transmural necrosis in CL-SBO patients. In line with this finding, higher ASA classification and old age were considered independent predictors of adverse outcomes for patients with acute intestinal ischemia (32). Masja et al. reached similar conclusions that patients with ASA classification ≥3 had a higher risk of irreversible perioperative ischemia (33).

Concerning imaging data, the increased unenhanced bowel-wall attenuation reveals potential diagnostic value among CL-SBO patients with transmural necrosis. This sign correlated with necrosis in CL-SBO with high specificity [100% (95% CI, 79–100)], and is thought to result from the extravasation of blood components due to capillary wall damage (20). Venous engorgement can lead to bowel wall thickening. However, various studies have different threshold values to define this characteristic, such as 3 mm (21) and 5 mm (34). Although the thickness of the involved bowel wall >3 mm was confirmed to be the risk factor for necrosis in our study, the systematic review of Millet et al. suggested that bowel wall thickening had decreased diagnostic value compared with increased unenhanced bowel-wall attenuation because of its lower sensitivity and specificity (22). The widespread engorgement of veins can contribute to the transudation of fluid across the serosa into the mesentery and peritoneal cavity (21). Carefully imaging and evaluating the mesentery and peritoneal cavity status may play a vital role in determining the ischemic severity of the affected intestinal segment. Patients with transmural necrosis are more likely to have signs of mesenteric fluid, diffuse mesenteric haziness and large peritoneal fluid in this study. The feces sign is typically found at the intestinal transition zone in patients with SBO. The results of the univariate analysis indicated that feces sign is significantly associated with necrosis. While univariate analysis indicated that the feces sign is significantly associated with necrosis, multivariate analysis did not support the feces sign as an independent predictor of transmural necrosis, consistent with the findings of Khaled et al. (27).

The blood circulation disorder caused by mechanical compression is regarded as the reason for ischemia in patients with CL-SBO. Most studies (2, 20, 21) have focused on the sign of engorgement in mesenteric vessels. In the current study, mesenteric venous engorgement did not demonstrate any value in differentiating transient ischemia from necrosis. However, careful clinical observation during this study, along with accumulated diagnostic experience, highlighted that the mesenteric vessel interruption occurred frequently in CL-SBO patients with necrotic intestinal segments — an observation previously unreported. This was further verified by the multivariate analysis data. This imaging biomarker, along with the ASA classification, demonstrated diagnostic efficacy in both group and subgroup analyses and was incorporated into a diagnostic model with excellent predictive ability for transmural necrosis in the external validation group (C-index = 0.89). Applying this model in the validation set showed that 93.33% of patients with both an ASA score >2 and mesenteric vessel interruption were diagnosed with transmural necrosis. In contrast, 90.91% of patients with neither a higher ASA score nor this innovative imaging sign were correctly excluded from the necrosis group. These high proportions underscore the signification role of mesenteric vessel interruption in resolving the challenging clinical problem of evaluating ischemia levels in CL-SBO.

The combination of unenhanced CT imaging and clinical features in our prediction model improved diagnostic accuracy compared with using unenhanced CT imaging features alone to predict bowel necrosis (17). Moreover, because the model relies on unenhanced CT imaging, it is suitable for patients who cannot use contrast agents. Compared with enhanced CT, this approach ensures diagnostic accuracy while being more convenient and having a broader clinical application range. For patients with bowel necrosis, inappropriate surgical strategies can lead to disease progression. Although laparoscopic surgery is becoming more common, intestinal resection remains a major reason for converting to open surgery (35). Irreversible intestine necrosis is likely to result in difficult-to-control septic shock, and laparoscopic surgery will significantly increase the risk associated with anesthesia. The incidence of postoperative complications and 30-day mortality is higher in patients requiring small intestinal resection than in those who underwent adhesiolysis alone. However, our prediction model guides the selection of surgical methods and help provide the best surgical strategy for patients.

Although the current study has a relatively large sample size and external test set, several limitations must be acknowledged. Firstly, as with all retrospective studies, selection bias is a concern. Secondly, while the value of the imaging marker of mesenteric vessel interruption has been verified in differentiating CL-SBO patients with necrosis from those with transient ischemia, this predictive marker has not yet been applied to distinguish between patients with simple SBO and SSBO. Its efficacy for this common clinical problem still needs to be investigated. Thirdly, the number of patients included in the validation set is limited and drawn from a single center. To establish the broader applicability of the diagnostic model and the new imaging marker, it is necessary to examine more CL-SBO patients across multiple centers. Fourthly, unenhanced CT, which does not require the injection of contrast agents, is relatively convenient. In emergency settings where medical resources are often scarce, unenhanced CT is generally preferred for patients with acute abdomen requiring CT examination, from a health economics perspective. Additionally, some patients may be allergic to contrast media or have conditions such as renal insufficiency or septic shock that make them unsuitable for enhanced CT. Therefore, while unenhanced CT is more convenient and efficient in emergencies, it is likely that diagnostic accuracy could be further improved if the model can be constructed using enhanced CT radiographic data. Fifthly, although CT data extraction was performed by two experienced radiologists, we did not quantitatively assess interobserver agreement. Finally, characteristics incorporated in building the logistic regression model are relatively simple, and more potential indicators must be explored.

## 5 Conclusion

After a rigorous statistical analysis, the ASA classification and CT appearance of mesenteric vessel interruption were found to be correlated and valuable in differentiating transmural necrosis from transient ischemia among CL-SBO patients. Utilizing this universal clinical index and innovative imaging marker, a diagnostic model was developed, yielding promising clinical results for predicting this perplexing clinical issue. The data shows potential for optimizing the diagnosis and treatment process for patients with SSBO. Implementing more efficient surgical strategies can improve the prognosis for CL-SBO patients with transmural necrosis.

## Data Availability

The original contributions presented in the study are included in the article/Supplementary material, further inquiries can be directed to the corresponding author.
