# Supplementary material for: A prediction model to differentiate transient ischemia from irreversible transmural necrosis in closed-loop small bowel obstruction
Source: Front Med (Lausanne). 2024 Sep 11;11:1466754. doi: 10.3389/fmed.2024.1466754 (PMC11422149; doi:10.3389/fmed.2024.1466754)
Supplement: Supplementary file 1 [file Data_Sheet_1.docx]

Supplementary Material

# Supplementary Figures and Tables

For more information on Supplementary Material and for details on the different file types accepted, please see [here](https://www.frontiersin.org/guidelines/author-guidelines#supplementary-material).

## Supplementary Figures


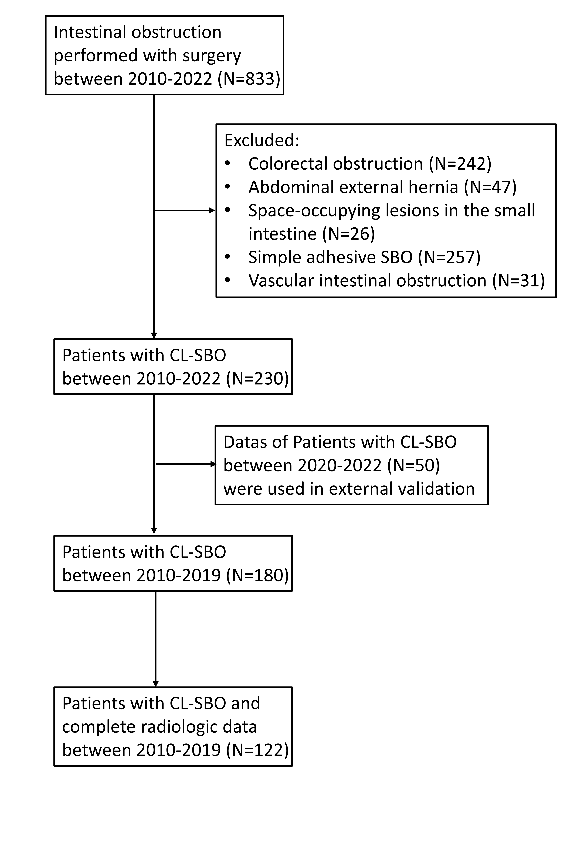


**Supplementary Figure 1.** The screening process for this study.


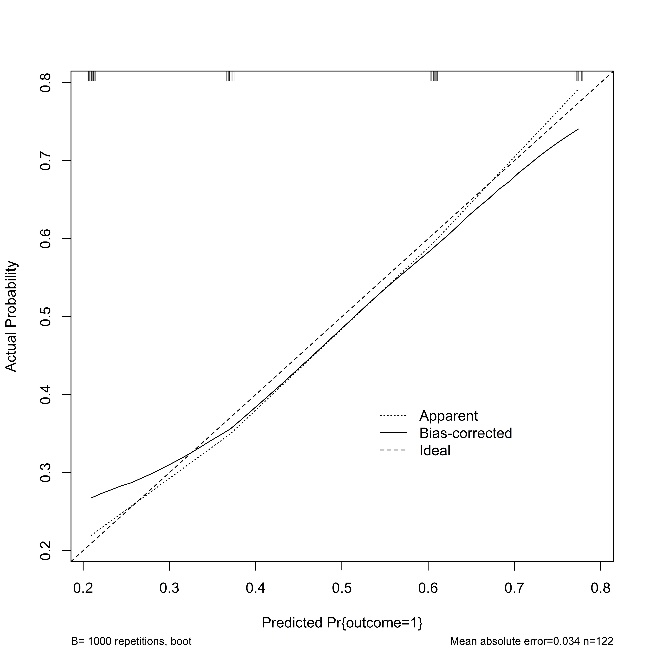


**Supplementary Figure 2.** The calibration curve of four tiers classified by ASA and Interruption of the mesenteric vessel.

## Supplementary Tables

**Supplementary Table A** Univariate analysis of clinical characteristics in total patients

| Variables | Transmural necrosis (n=96) | Transient ischemia (n=84) | *p* value |
| --- | --- | --- | --- |
| Advanced age(y),n(%) |  |  | **0.038** |
| Yes | 56 (58.3) | 36 (42.9) |  |
| No | 40 (41.7) | 48 (57.1) |  |
| Sex |  |  | 0.156 |
| Male, n (%) | 49 (51) | 34 (40.5) |  |
| Female, n (%) | 47 (49) | 50 (59.5) |  |
| ASA score, n (%) |  |  | **0.004** |
| Class 1 or 2 | 52 (54.2) | 63 (75) |  |
| Class 3 or more | 44 (45.8) | 21 (25) |  |
| BMI | 21.7 ± 3.6 | 21.5 ± 3.6 | 0.636 |
| NLR | 9.7 (5.7-14.4) | 7.8 (4.0-12.3) | **0.044** |
| WBC(10^9/L) | 10.14 (6.9-13.9) | 9.8 (6.9-13.4) | 0.420 |
| Neutrophils percentage (%) | 85.8 (78.4-89.5) | 81.5 (74.4-88.6) | 0.052 |
| Lymphocyte percentage (%) | 8.9 (6.1-13.7) | 10.5 (7.0-17.5) | **0.045** |
| From onset to operation (h) | 48 (24-168) | 48 (24-168) | 0.646 |
| Highest temperature (℃) | 36.9 (36.5-37.2) | 37 (36.6-37.2) | 0.852 |
| Abdominal pain, n (%) |  |  | 0.499 |
| With | 94 (97.9) | 84 (100) |  |
| Without | 2 (2.1) | 0 (0) |  |
| Emesis, n (%) |  |  | 0.511 |
| Positive | 69 (71.9) | 64 (76.2) |  |
| Negative | 27 (28.1) | 20 (23.8) |  |
| Exhaust or defecation, n (%) |  |  | 0.632 |
| Yes | 20 (20.8) | 20 (23.8) |  |
| No | 76 (79.2) | 64 (76.2) |  |
| Peritoneal irritation, n (%) |  |  | 0.577 |
| Positive | 37 (38.5) | 29 (34.5) |  |
| Negative | 59 (61.5) | 55 (65.5) |  |
| History of abdominal  surgery or infection, n (%) |  |  | 0.261 |
| Absent | 35 (36.5) | 24 (28.6) |  |
| Present | 61 (63.5) | 60 (71.4) |  |
| SIRS, n (%) |  |  | 0.129 |
| Positive | 39 (40.6) | 25 (29.8) |  |
| Negative | 57 (59.4) | 59 (70.2) |  |
| Preoperative stomach tube placement, n (%) |  |  | 0.610 |
| Yes | 39 (40.6) | 31 (36.9) |  |
| No | 57 (59.4) | 53 (63.1) |  |
| Preoperative antibiotic  usage, n (%) |  |  | 0.968 |
| Yes | 50 (52.1) | 44 (52.4) |  |
| No | 46 (47.9) | 40 (47.6) |  |
| Causes of SSBO, n (%) |  |  | 0.290 |
| Fibrous cord or  bundle-like structure | 27 (28.1) | 25 (29.8) |  |
| Torsion | 10 (10.4) | 15 (17.9) |  |
| Internal hernia | 59 (61.5) | 44 (52.4) |  |

Advanced age, age over 61; ASA, American Society of Anesthesiologists; BMI, Body Mass Index; NLR, neutrophil lymphocyte ratio; WBC, white blood cells; SIRS, systemic inflammatory response syndrome; SSBO, strangulated small bowel obstruction.

Continuous variates were presented as median (IQR) based on Mann‐Whitney U test; categorical variates were presented as n (%) with χ2 or fisher exact test unless otherwise noted.

**Supplementary Table B** Logistic regression for significant clinical indicators in total population

| Variables |  | Multivariate analysis |  |
| --- | --- | --- | --- |
|  |  | Odds Ratio (95%CI) | *p* value |
| Advanced age (y) |  | 1.293 (0.650-2.574) | 0.464 |
| ASA score |  | 2.103 (1.005-4.401) | **0.049** |
| NLR |  | 1.012 (0.969-1.057) | 0.580 |
| Lymphocyte percentage |  | 0.997 (0.954-1.041) | 0.878 |

Advanced age, age over 61; ASA, American Society of Anesthesiologists; NLR, neutrophil lymphocyte ratio; CI, Confidence Interval.

**Supplementary Table C** Univariate analysis of clinical characteristics in subgroup

| Variables | Transmural necrosis (n=57) | Transient ischemia (n=65) | *p* value |
| --- | --- | --- | --- |
| Advanced age (y), n (%) |  |  | 0.097 |
| Yes | 34 (59.6) | 29 (44.6) |  |
| No | 23 (40.4) | 36 (55.4) |  |
| Sex |  |  | 0.893 |
| Male, n (%) | 30 (52.6) | 35 (53.8) |  |
| Female, n (%) | 27 (47.4) | 30 (46.2) |  |
| ASA score, n (%) |  |  | **0.041** |
| Class 1 or 2 | 31 (54.4) | 47 (72.3) |  |
| Class 3 or more | 26 (45.6) | 18 (27.7) |  |
| BMI | 22.5(19.6-24.4) | 21.3(18.9-24.1) | 0.220 |
| NLR | 10.8 (6.5-14.3) | 8.0 (4.0-14.5) | 0.095 |
| WBC(10^9/L) | 10.2 (7.2-14.7) | 10.5 (7.0-13.4) | 0.446 |
| Neutrophils percentage(%) | 86.5 (79.9-89.2) | 81.7 (74.1-90.1) | 0.199 |
| Lymphocyte percentage(%) | 8.2 (6.2-12.2) | 10.3 (6.2-17.9) | 0.093 |
| From onset to operation (h) | 48 (24-168) | 48 (24-144) | 0.512 |
| Highest temperature (℃) | 36.9 (36.6-37.2) | 37 (36.6-37.2) | 0.998 |
| Abdominal pain, n (%) |  |  | 0.467 |
| With | 56 (98.2) | 65 (100) |  |
| Without | 1 (1.8) | 0 (0) |  |
| Emesis, n (%) |  |  | 0.527 |
| Positive | 41 (71.9) | 50 (76.9) |  |
| Negative | 16 (28.1) | 15 (23.1) |  |
| Exhaust or defecation, n (%) |  |  | 0.301 |
| Yes | 12 (21.1) | 19 (29.2) |  |
| No | 45 (78.9) | 46 (70.8) |  |
| Peritoneal irritation, n (%) |  |  | 0.885 |
| Positive | 20 (35.1) | 22 (33.8) |  |
| Negative | 37 (64.9) | 43 (66.2) |  |
| SIRS, n (%) |  |  | 0.639 |
| Positive | 18 (31.6) | 18 (27.7) |  |
| Negative | 39 (68.4) | 47 (72.3) |  |
| Preoperative stomach tube  placement, n (%) |  |  | 0.746 |
| Yes | 20 (35.1) | 21 (32.3) |  |
| No | 37 (69.4) | 44 (67.7) |  |
| Preoperative antibiotic  usage, n (%) |  |  | 0.364 |
| Yes | 31 (54.4) | 30 (46.2) |  |
| No | 26 (45.6) | 35 (53.8) |  |
| Causes of SSBO, n (%) |  |  | 0.152 |
| Fibrous cord or  bundle-like structure | 9 (15.8) | 16 (24.6) |  |
| Torsion | 6 (10.5) | 12 (18.5) |  |
| Internal hernia | 42 (73.7) | 37 (56.9) |  |
|  |  |  |  |

Advanced age, age over 62; ASA, American Society of Anesthesiologists; BMI, Body Mass Index; NLR, neutrophil lymphocyte ratio; WBC, white blood cells; SIRS, systemic inflammatory response syndrome; SSBO, strangulated small bowel obstruction.

Continuous variates were presented as median (IQR) based on Mann‐Whitney U test; categorical variates were presented as n (%) with χ2 or fisher exact test unless otherwise noted.

**Supplementary Table D** Comparison of CT findings in subgroup

| Variables | Transmural necrosis (n=57) | Transient ischemia (n=65) | *p* value |
| --- | --- | --- | --- |
| Increased unenhanced bowel-wall attenuation, n (%) |  |  | **0.003** |
| Positive | 15 (26.3) | 4 (6.2) |  |
| Negative | 42 (73.7) | 61 (93.8) |  |
| Bowel wall thickening, n (%) |  |  | **<0.001** |
| Positive | 37 (64.9) | 21 (32.3) |  |
| Negative | 20 (35.1) | 44 (67.7) |  |
| Bowel loop dilatation, n (%) |  |  | 1 |
| Positive | 54 (94.7) | 62 (95.4) |  |
| Negative | 3 (5.3) | 3 (4.6) |  |
| Diameter of bowel loop | 3.6 (3.0-4.1) | 3.7 (3.1-4.3) | 0.449 |
| Mesenteric fluid, n (%) |  |  | **0.007** |
| Positive | 47 (82.5) | 39 (60) |  |
| Negative | 10 (17.5) | 26 (40) |  |
| Mesenteric haziness, n (%) |  |  | **0.017** |
| Absent | 2 (3.5) | 7 (10.8) |  |
| Focal | 17 (29.8) | 28 (43.1) |  |
| Diffuse | 38 (66.7) | 30 (46.2) |  |
| Mesenteric venous engorgement, n (%) |  |  | 0.339 |
| Positive | 25 (43.9) | 23 (35.4) |  |
| Negative | 32 (56.1) | 42 (64.6) |  |
| Radial distribution, n (%) |  |  | 0.281 |
| Positive | 31 (54.4) | 29 (44.6) |  |
| Negative | 26 (45.6) | 36 (55.4) |  |
| Pneumatosis intestinalis, n (%) |  |  | 0.467 |
| Positive | 1 (1.8) | 0 (0) |  |
| Negative | 56 (98.2) | 65 (100) |  |
| Pneumoperitoneum, n (%) |  |  | 0.467 |
| Positive | 1 (1.8) | 0 (0) |  |
| Negative | 56 (98.2) | 65 (100) |  |
| Peritoneal fluid, n (%) |  |  | **0.039** |
| Absent | 6 (10.5) | 13 (20) |  |
| Small | 37 (64.9) | 44 (67.7) |  |
| Large | 14 (24.6) | 8 (12.3) |  |
| Whirl sign, n (%) |  |  | 0.251 |
| Positive | 7 (12.3) | 13 (20) |  |
| Negative | 50 (87.7) | 52 (80) |  |
| Feces sign, n (%) |  |  | **0.033** |
| Absent | 33 (57.9) | 45 (69.2) |  |
| FS1 | 14 (24.6) | 6 (9.2) |  |
| FS2 | 10 (17.5) | 10 (15.4) |  |
| FS3 | 0 (0) | 4 (6.2) |  |
| Degree of obstruction, n (%) |  |  | 0.415 |
| Negative | 1 (1.8) | 1 (1.5) |  |
| Low | 14 (24.6) | 12 (18.5) |  |
| High | 42 (73.7) | 52 (80) |  |
| Interruption of the mesangial vessel, n (%) |  |  | **<0.001** |
| Positive | 41 (71.9) | 20 (30.8) |  |
| Negative | 16 (28.1) | 45 (69.2) |  |

Continuous variates were presented as median (IQR) based on Mann‐Whitney U test; categorical variates were presented as n (%) with χ2 or fisher exact test unless otherwise noted.
